# Influence of Tramadol on the Intravenous Pharmacokinetics of Dipyrone Active Metabolites in Dogs

**DOI:** 10.1111/jvp.70073

**Published:** 2026-04-24

**Authors:** Andressa Nunes Mouta, Kathryn Nóbrega Arcoverde, Robson dos Anjos Honorato, Naftáli Silva Fernandes, Caio Vinicius Almeida de Oliveira, Glícia Cavalcante Mesquita, Yanna Deysi Bandeira Passos, Gabriel Araújo‐Silva, Valéria Veras de Paula

**Affiliations:** ^1^ Departamento de Ciência Animal Universidade Federal Rural Do Semi‐Árido (UFERSA) Mossoró Brazil; ^2^ Centro Universitário INTA – UNINTA Rua Antônio Rodrigues Magalhães, 359, Dom Expedito Lopes Sobral Brazil; ^3^ Escola de Química Universidade Do Estado Do Amapá, Avenida Presidente Vargas, Centro Macapá Brazil

**Keywords:** 4‐aminoantipyrine, 4‐methylaminoantipyrine, metamizole, N‐desmethyltramadol, O‐desmethyltramadol

## Abstract

This study aimed to compare the pharmacokinetic profile of the active dipyrone metabolites 4‐methylaminoantipyrine (MAA) and 4‐aminoantipyrine (AA) administered alone and in combination with tramadol in dogs. Nine mixed‐breed dogs, weighing 15.33 ± 2.25 kg, participated in a two‐treatment crossover design: G1 received intravenous dipyrone (25 mg kg^−1^) and G2 received dipyrone (25 mg kg^−1^) combined with tramadol (2 mg kg^−1^), with a 15‐day washout. Blood samples were collected up to 48 h and plasma concentrations analyzed using UPLC–MS/MS. Pharmacokinetic parameters were calculated using PKSolver 2.0. Normally distributed data were analyzed with the *t*‐test, and non‐normal data with the Mann–Whitney test (*p* < 0.05); parameters with significant differences were subjected to Pearson correlation. Analyses were performed using Python. For MAA, Cmax and C0 differed significantly. For AA, *AUC*_0–*t*_, *AUC*_0→∞_, Cl, and *MRT*_0→∞_ differed significantly. Tramadol and its metabolites were also described. The pharmacokinetic interaction between dipyrone and tramadol increased systemic exposure (AUC) of AA and inhibited the conversion of tramadol to M1, without direct changes in the severe adverse effects associated with MAA. Further studies involving nociceptive stimuli and multiple‐dose regimens are needed to assess clinical relevance.

## 1 Introduction

Dipyrone, also known as metamizole, is an analgesic compound with antipyretic and spasmolytic properties, belonging to the pyrazolone derivatives (Schütter et al. 2024; Garcia Del Campo et al. 2021). Its mechanism of action involves interference with prostaglandin synthesis through the reduction of the oxidative state of the cyclooxygenase (COX) enzyme, acting on both COX‐1 and COX‐2. However, COX‐3 is considered the most sensitive isoform, primarily located in the central nervous system and derived from COX‐1 (Chandrasekharan et al. 2002). Opioidergic pathways have also been reported as part of the drug's mechanism of action (Tortorici et al. 1996; Jasiecka et al. 2014).

The clinical use of dipyrone in small animals is significant in some South American countries, including Brazil. For a long time, this compound was classified as a nonsteroidal anti‐inflammatory drug; however, recent classifications define it as a non‐opioid analgesic. Regarding its metabolism, dipyrone is known to be a prodrug that is rapidly chemical‐hydrolyzed in plasma into its main metabolite, 4‐methylaminoantipyrine (MAA) (Levy et al. 1995; Bachmann et al. 2022). MAA is an active metabolite and undergoes further metabolism to form a second active metabolite, 4‐aminoantipyrine (AA), via N‐demethylation, and an inactive metabolite, 4‐formylaminoantipyrine (FAA), via C‐oxidation (Levy et al. 1995; Lutz 2019; Bachmann et al. 2022). Additionally, AA may undergo acetylation to produce another inactive metabolite, 4‐acetylaminoantipyrine (AAA) (Lutz 2019). Pharmacokinetic studies investigating the use of dipyrone alone have been previously reported (Kalchofner et al. 2015; Giorgi et al. 2018; Mouta et al. 2025).

Tramadol is an opioid analgesic widely used for pain management in humans and animals, with central nervous system activity (Subedi et al. 2019; Ruel and Steagall 2019). This drug is classified as an atypical opioid, as it produces analgesia through μ‐receptor activity and exhibits a monoaminergic action by inhibiting the reuptake of norepinephrine and serotonin (Grond and Sablotzki 2004; Bravo et al. 2017). Studies evaluating tramadol in dogs for the management of clinical and postoperative conditions are well documented in the literature (Mansfield and Beths 2015; Donati et al. 2021).

Regarding tramadol pharmacokinetics, it is metabolized by cytochrome P450 enzymes in humans (CYP3A4 and CYP2D6) and in dogs (CYP2D15 for M1, CYP2B11, CYP3A12, and CYP2C41 for M2). Its primary active metabolite, O‐desmethyltramadol (M1), is responsible for most of its analgesic effect (Miotto et al. 2017). N‐desmethyltramadol (M2) is an inactive metabolite, whereas N,O‐didesmethyltramadol (M5), formed from M1 metabolism, is also active (Beakley et al. 2015; Domínguez‐Oliva et al. 2021).

Pharmacokinetic studies of tramadol administered alone in dogs are extensively discussed in the literature due to inconsistencies regarding the detection and minimum concentrations of M1 required to produce analgesia (Kukanich and Papich 2004; McMillan et al. 2008; Giorgi et al. 2010; Vettorato et al. 2010; Kukanich and Papich 2011).

The combination of opioid and non‐opioid analgesics may result in additive or complementary effects in animals, improving analgesic efficacy while minimizing adverse effects through pharmacological synergy (Moreno‐Rocha et al. 2016). International pain management guidelines from the World Small Animal Veterinary Association (WSAVA, 2022) and the American Animal Hospital Association (AAHA, 2022) emphasize the use of multimodal analgesic strategies to optimize pain control in both dogs and cats. Within this framework, tramadol is cited as an adjunctive pharmacological option in multimodal protocols, with particular relevance in cats and a more variable and limited efficacy in dogs, and is discussed especially in clinical settings where access to other controlled analgesics is limited or contraindicated.

Although dipyrone is not explicitly addressed in these international guidelines, it is widely used in veterinary clinical practice in several countries, including Brazil, particularly for the management of acute and perioperative pain in dogs and cats. This practice is supported by a national survey on veterinary prescribing patterns in Brazil, in which 79% of respondents reported the use of tramadol and 33% reported the use of metamizole for pre‐ and postoperative pain management in routine surgical procedures, such as ovariohysterectomy (Lorena et al. 2014). Clinical studies investigating the combination of dipyrone and tramadol in dogs have been reported for pain management in oncologic patients (Flôr et al. 2013; Teixeira et al. 2013). However, despite their extensive clinical use, the pharmacokinetic profile of this combination has not yet been described in dogs.

In this context, the objective of the present study was to characterize the pharmacokinetic profile of dipyrone when administered in combination with tramadol in dogs, with emphasis on the formation of its primary active metabolites, 4‐methylaminoantipyrine (MAA) and 4‐aminoantipyrine (AA). Additionally, the production of tramadol metabolites‐O‐desmethyltramadol (M1), N‐desmethyltramadol (M2), and N,O‐didesmethyltramadol (M5)—was evaluated following intravenous administration of 25 mg kg^−1^ dipyrone and 2 mg kg^−1^ tramadol using ultra‐performance liquid chromatography coupled to mass spectrometry (UPLC–MS/MS). Our hypothesis is that tramadol may interfere with dipyrone hepatic metabolism through enzymatic competition, resulting in alterations in the concentrations of its active metabolites, possibly with a longer *T*½ of the drugs.

## 2 Materials and Methods

### 2.1 Animals and Study Design

The study was approved by the Animal Use Ethics Committee of the Federal Rural University of the Semi‐Arid (CEUA/UFERSA), under protocol number 07/2023 (May 2, 2023). Animals were included in the study by convenience after undergoing clinical and laboratory examinations. A total of 10 animals were initially enrolled; however, one animal was excluded from statistical analysis due to inconsistencies in its results. Nine dogs (5 males and 4 females), weighing 15.33 ± 2.25 kg, healthy, intact, aged between 1 and 5 years (1.44 ± 0.88), and of mixed breed, were included. Informed consent and authorization for participation were obtained from the animals' owners.

The animals were grouped in kennels on the premises of UFERSA's Internal Veterinary Medicine Laboratory and subjected to 8‐h food and 4‐h water fasts. All dogs underwent physical examination and laboratory tests (complete blood count and serum biochemistry—urea, creatinine, total proteins, albumin, alanine aminotransferase, and alkaline phosphatase). Only dogs confirmed to be healthy were included in the study.

The nine dogs participated in a two‐treatment crossover design. In group G1, they received 25 mg kg^−1^ dipyrone (D‐500, Zoetis, São Paulo, Brazil) intravenously, while in group G2, the same animals received 25 mg kg^−1^ dipyrone (D‐500, Zoetis, São Paulo, Brazil) combined with 2 mg kg^−1^ tramadol (Tramadol Hydrochloride, Teuto, Anápolis, Goiás, Brazil), also administered intravenously. The washout period between treatments was 15 days.

For drug administration, animals were manually restrained, and the cephalic vein region underwent trichotomy and antisepsis for insertion of two 20 G catheters, each connected to a 3‐way stopcock. One access was used exclusively for drug administration, while the other was dedicated to blood sampling to avoid cross‐contamination. Drugs were administered over 2 min using two separate infusion pumps (DigiPUMP SR 31X—Digicare) through the same administration venous access.

All animals were monitored for any sign of behavioral changes and adverse effects, including salivation, muscle spasms, restlessness, and tachypnea. The presence, onset, and duration of these effects were recorded.

### 2.2 Pharmacokinetic Study

To determine plasma concentrations of MAA, AA, tramadol, M1, M2, and M5, 4 mL of blood were collected from the cephalic vein of all animals, at the following time points: 0 (prior to drug administration), and 5, 15, 30, 45 min, 1, 1.5, 2, 4, 6, 8, 10, 12, 24, 36, and 48 h. Blood samples were collected through a separate venous access, which was flushed with heparinized saline solution after each collection to ensure patency. The samples were stored in tubes containing ethylenediaminetetraacetic acid (EDTA), centrifuged at 3500 rpm for 10 min to obtain plasma, which was then stored in cryogenic tubes at −80°C until analysis by ultra‐performance liquid chromatography coupled with mass spectrometry (UPLC‐MS/MS).

### 2.3 Sample Extraction Procedures

Aliquots of the obtained plasma samples (250 μL) were mixed with 10 μL of 0.1 mg mL^−1^ metoprolol solution (internal standard) and 800 μL of acetonitrile, followed by vortexing homogenization for 60 s and centrifugation for 10 min at 10,000 rpm. The supernatants (900 μL) were transferred to vials and 5 μL was injected into the UPLC–MS/MS chromatographic system, consisting of a Nexera 2 UPLC coupled to an LCMS‐8040 mass spectrometer (Shimadzu, Japan) and a Phenomenex UPLC Luna Ômega C18 column (1.6 μm, 2.1 × 50 mm) (Phenomenex, Torrance, CA, USA).

### 2.4 UPLC‐MS/MS Analytical Conditions

The mobile phase consisted of acetonitrile and 0.1% formic acid solution (75:25, v/v) at 0.3 mL·min^−1^. The run time was set at 2.0 min and the injected sample volume was 5.0 μL. The column temperature was adjusted to 40°C, and the autosampler was set to 5°C. For dipyrone metabolites (MAA, AA) and tramadol with its metabolites (M1, M2, M5), the mass spectrometer was operated in multiple reaction monitoring (MRM) mode in positive electrospray ionization (ESI+). The applied collision energy and cone voltage were 28 and 25 V, respectively. The nebulizer gas flow rate was 3 L min^−1^, using argon as the collision gas at a flow rate of 15 L min^−1^. The mass spectrometer was set to monitor the band transition of parent and daughter ions with a dwell time of 0.1 s. MRM data were acquired and analyzed using the Labsolution software (Shimadzu, Japan).

The mass and charge ratio (m/z) of the main analyzed ions were: MAA (218.10), AA (204.10), tramadol (264.70), M1 (250.20), M2 (250.30), M5 (236.40), and metoprolol (268.20); while the daughter ions were: MAA (159.10), AA (77.00), tramadol (58.10), M1 (58.10), M2 (232.17), M5 (107.42), and metoprolol (131.00).

### 2.5 Validation

The analytical method was revalidated following the guidelines of the International Conference on Harmonization (ICH, 2018) and National Health Surveillance Agency criteria (ANVISA 2017). A drug‐free plasma was spiked with a standard solution to construct calibration curves. Quality control samples (spots) were prepared at low (1600 ng/mL), medium (5600 ng/mL), and high (40,000 ng/mL) concentrations for MAA and AA; and low (10 ng/mL), medium (500 ng/mL), and high (4000 ng/mL) concentrations for tramadol and its metabolites. For tramadol and its metabolites, the calibration range was 1–5000 ng/mL, and for MAA and AA, it was 800–40,000 ng/mL. These samples were used to determine absolute recovery and intra‐ and inter‐day precision. Selectivity was evaluated by preparing the lower limit of quantification (LLOQ), set at 0.076 μg/mL for MAA and AA and 0.8 ng/mL for tramadol and its metabolites in drug‐free plasma. Stability was assessed for the biological matrix stored at −70°C, at room temperature (20°C), and in samples processed in the autosampler. M2 and M5, due to their similar molecular weights to M1, were quantified as M1 equivalents.

### 2.6 Pharmacokinetic Analysis

Pharmacokinetic parameters were calculated applying a non‐compartmental model with PKSolver software (Zhang et al. 2010). The observed variables were as follows: terminal phase rate constant (*λz*), half‐life (*T*½), maximum plasma concentration (Cmax), plasma concentration at time zero (C0), time to reach Cmax (Tmax), area under the plasma concentration curve from zero to the time of the last measurable concentration (*AUC*_0→*t*_), the extrapolation of AUC to infinity (*AUC*_0→∞_), the ratio between *AUC*_0→*t*_ to *AUC*_0→∞_, the mean residual time from moment zero to infinity (*MRT*_0→∞_), volume of distribution (Vd), which is the estimated volume of distribution based on the AUC, volume of distribution at steady state (Vss), and the clearance (Cl).

### 2.7 Statistical Analysis

Statistical power was assessed using effect size estimation (Cohen's d) for key pharmacokinetic variables (Cmax, *AUC*_0→∞_, and Cl_obs) to evaluate the sensitivity of the experimental design. Results indicated that, although the sample size per group (*n* = 9) did not provide ideal power for all variables, it was sufficient to detect significant differences (> 80% for *AUC*_0→∞_). Normality was evaluated for the specified parameters. Normally distributed data were analyzed using the *t*‐test, while non‐normal data were assessed using the Mann–Whitney test, with *p* < 0.05 considered statistically significant. Pearson correlation was performed for all parameters with significant differences. Linear Pearson correlation was applied to parameters showing statistical differences for AA. Statistical analyses were conducted using Python.

## 3 Results

The method was revalidated to ensure data accuracy according to previous studies (De Macêdo et al. 2021; Arcoverde et al. 2023; Araújo‐Silva et al. 2024). All calibration curves presented R^2^ greater than 0.99, demonstrating linearity between equipment responses and curve concentrations. The method also exhibited satisfactory selectivity, reproducibility, and repeatability, with relative standard deviations below 5%. The samples were stable under the conditions used for analysis.

**FIGURE 1:**
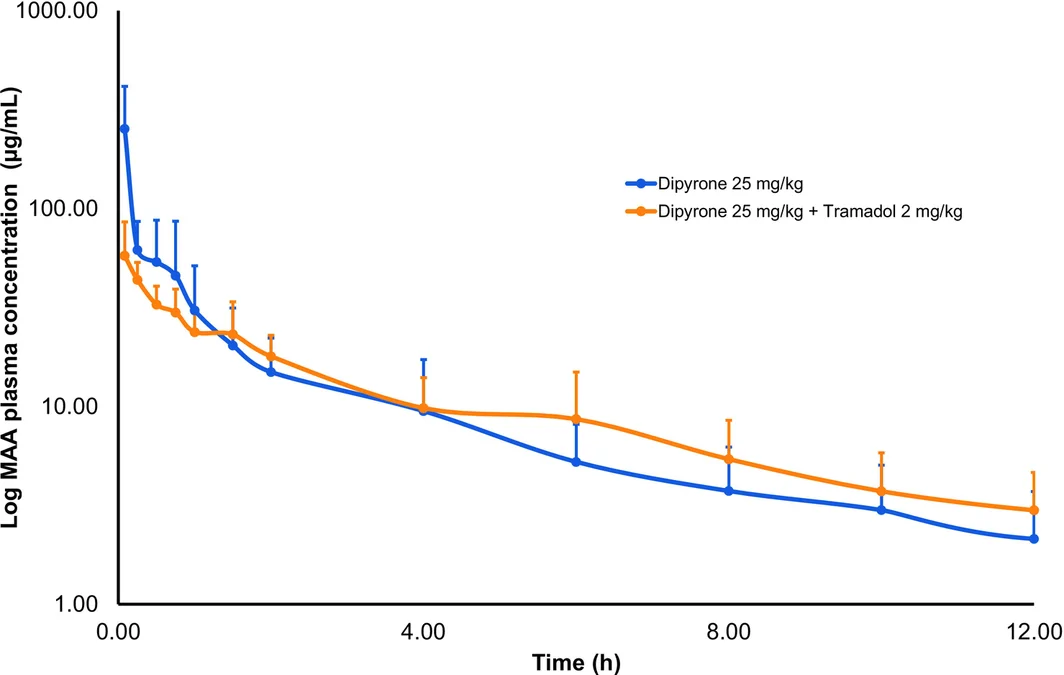
Log of the plasma concentration vs. time for 4‐methylaminoantipyrine (MAA) following intravenous administration of dipyrone (25 mg kg^−1^) alone and in association with tramadol (2 mg kg^−1^) in nine healthy dogs.

**FIGURE 2:**
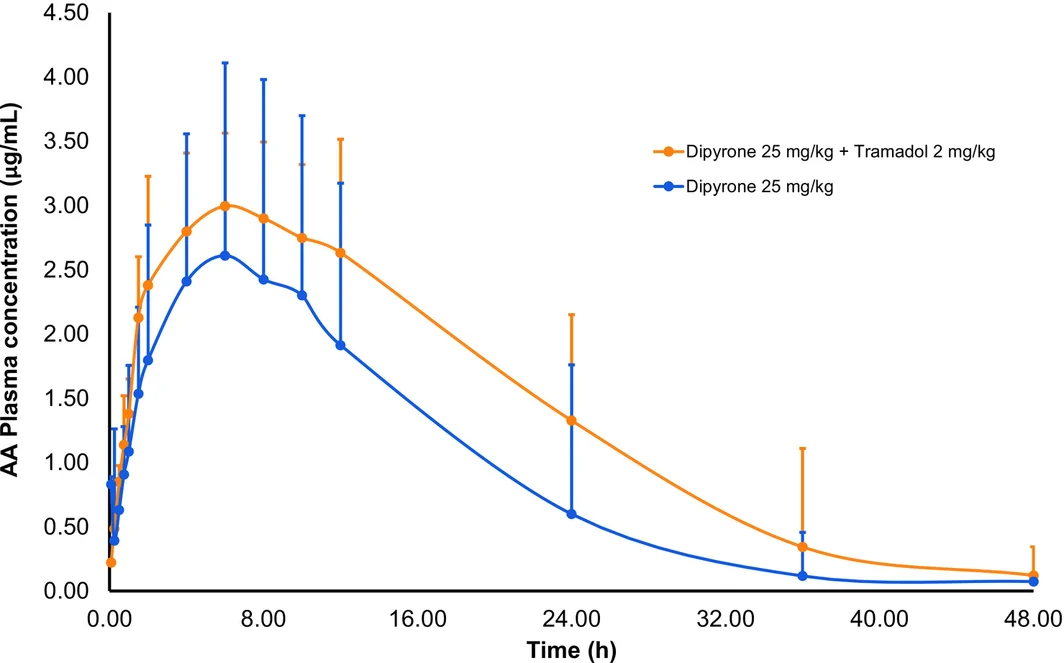
Plasma concentration vs. time for 4‐aminoantipyrine (AA) following intravenous administration of dipyrone (25 mg kg^−1^) alone and in association with tramadol (2 mg kg^−1^) in nine healthy dogs.

**FIGURE 3:**
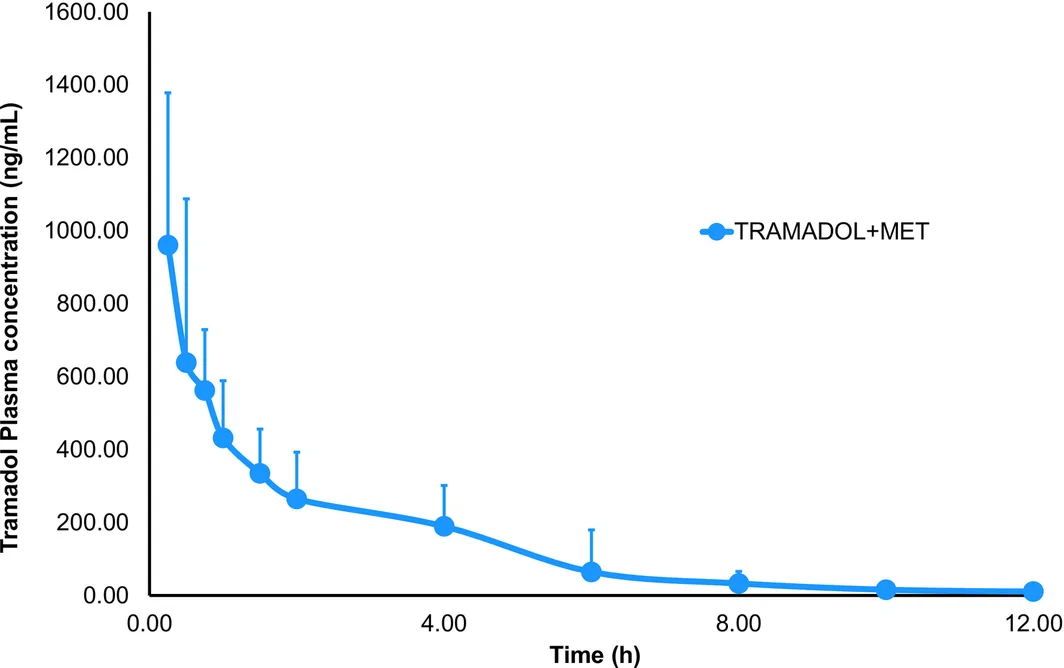
Plasma concentration vs. time for tramadol (2 mg kg^−1^) following intravenous administration with dipyrone (25 mg kg^−1^) in nine healthy dogs.

**FIGURE 4:**
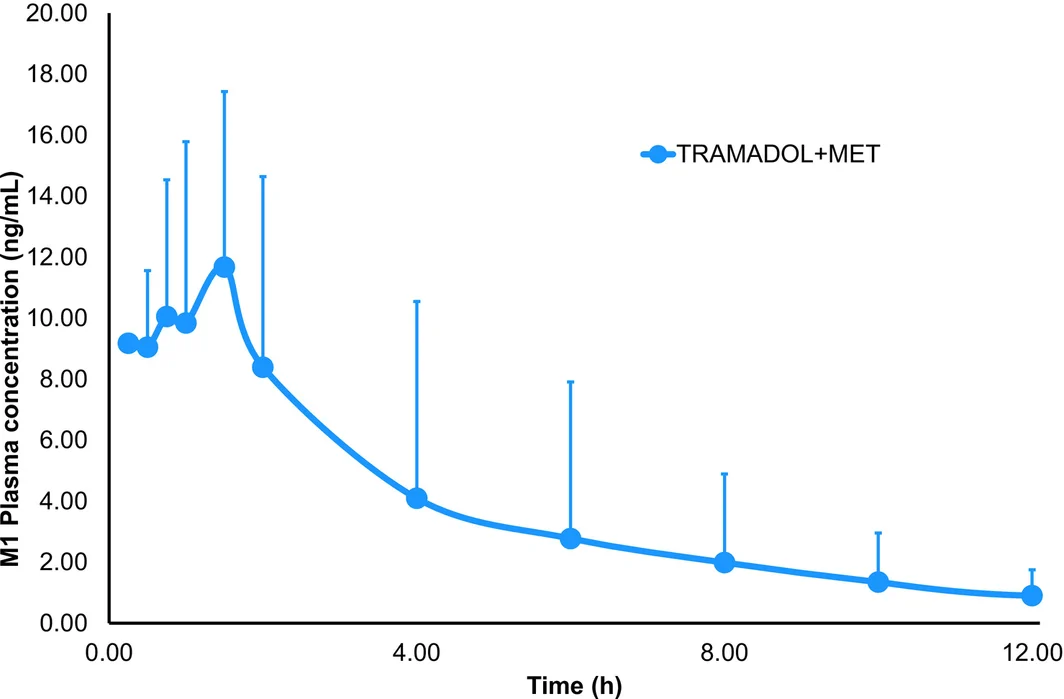
Plasma concentration vs. time for O‐desmethyltramadol (M1) (2 mg kg^−1^) following intravenous administration with dipyrone (25 mg kg^−1^) in nine healthy dogs.

After intravenous administration of dipyrone and dipyrone‐tramadol, metabolites were monitored for 48 h in all animals (Figures 1, 2, 3, 4). In G1, MAA was detectable and quantifiable for 48 h in all animals; AA was detectable and quantifiable for 24 h in seven animals. In G2, MAA was quantifiable for 48 h in three animals and detectable for 48 h in seven animals, whereas AA was quantifiable for 24 h in six animals and detectable for 48 h in all nine animals. Tramadol was detectable and quantifiable for 24 h in all animals; M1 was detectable for 48 h in four animals and quantifiable for 12 h in two animals; M2 was detectable for 36 h in six animals and quantifiable for 10 h in one animal; M5 was detectable for 10 h in all animals and quantifiable for 4 h in one animal.

**FIGURE 5:**
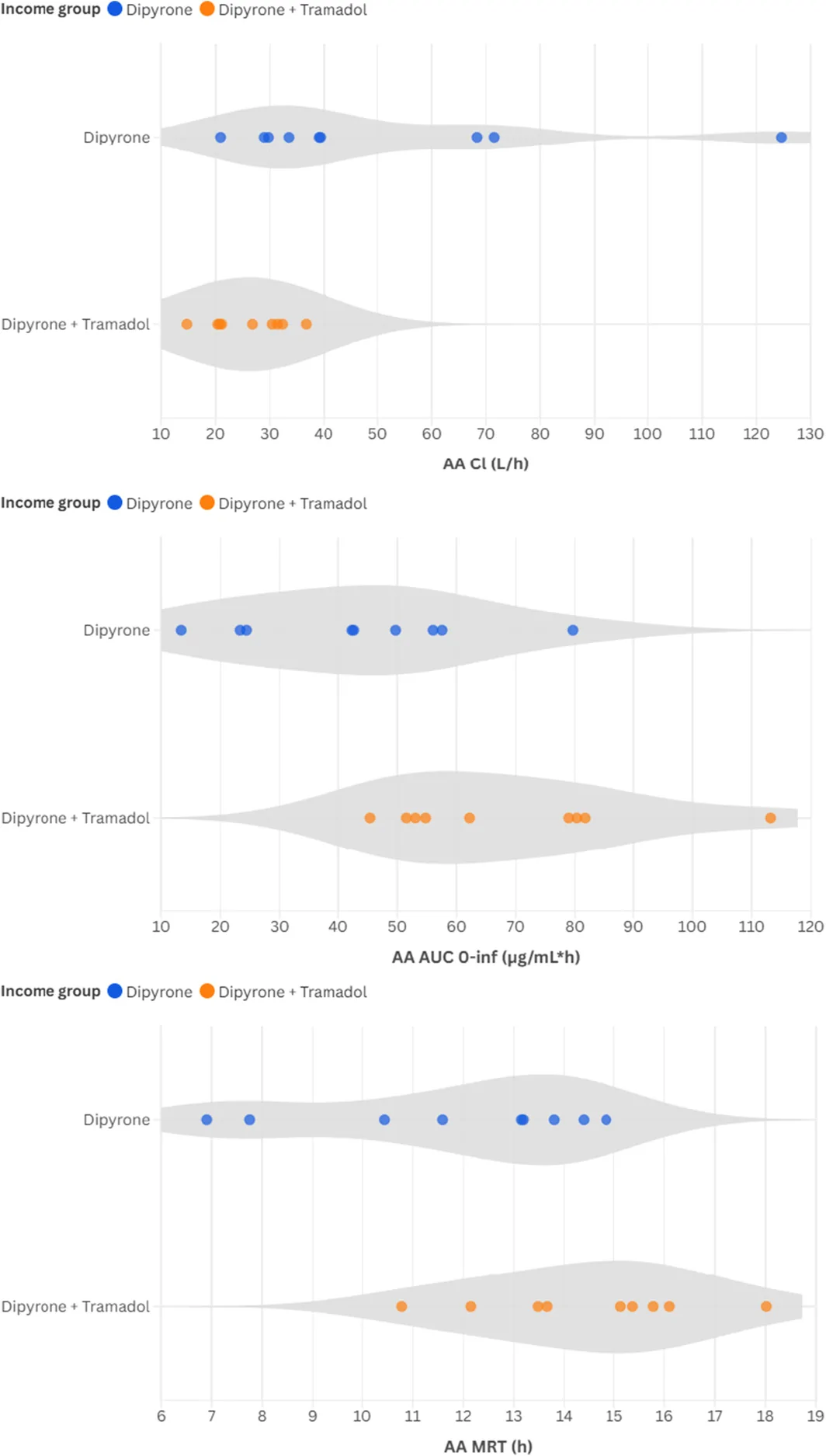
AA variables showing statistically significant differences between the dipyrone group (G1) and the dipyrone plus tramadol group (G2). (A) Cl (L/h); (B) *AUC*_0→∞_ (μg/mL h); (C) *MRT*_0→∞_ (h).

Regarding the pharmacokinetic parameters analyzed, the data are presented in the tables below. Table 1 shows the results for the metabolite MAA, which exhibited statistically significant differences in Cmax and C0.

**TABLE 1:** Pharmacokinetic parameters of 4‐methylaminoantipyrine (MAA) following intravenous administration of 25 mg kg^−1^ dipyrone (G1) and 25 mg kg^−1^ dipyrone combined with 2 mg kg^−1^ tramadol (G2) in dogs (*n* = 9).

MAA		G1 Dipyrone 25 mg kg^−1^		G2 Dipyrone 25 mg kg^−1^ + Tramadol 2 mg kg^−1^	
Parameters	Unit	Mean ± SD	Median (min–max)	Mean ± SD	Median (min–max)
Lambda_z (*λz*)	1/h	0.07 ± 0.09	0.04 (0.02–0.29)	0.05 ± 0.05	0.03 (0.02–0.18)
*T*½	H	19.53 ± 13.18	15.44 (2.35–38.58)	24.06 ± 12.85	25.57 (3.73–38.50)
Cmax	μg/mL	233.52 ± 161.32*	171.72 (71.78–548.74)	59.62 ± 24.92*	60.95 (30.08–115.67)
C0	μg/mL	481.24 ± 558.14*	303.60 (77.45–1912.98)	73.00 ± 36.43*	66.60 (35.84–160.40)
Tmax	H	—		—	
*AUC*_0→*t*_	μg/mL*h	216.21 ± 114.91	183.75 (67.37–404.65)	179.31 ± 51.60	160.94 (116.25–259.05)
*AUC*_0→∞_	μg/mL*h	240.60 ± 135.29	202.76 (70.00–441.35)	206.41 ± 63.49	185.10 (119.92–293.63)
*AUC*_0→*t*_/_0→∞_		0.92 ± 0.07	0.91 (0.76–0.99)	0.88 ± 0.06	0.88 (0.79–0.96)
*MRT*_0→∞_	H	12.25 ± 9.36	9.48 (2.02–32.43)	18.21 ± 7.85	18.68 (4.47–26.24)
Vd	(L)	208.54 ± 122.76	194.84 (70.91–908.30)	276.84 ± 143.00	305.85 (74.95–482.60)
Cl	(L/h)	10.22 ± 7.53	8.22 (3.73–23.81)	8.78 ± 2.69	9.00 (5.67–13.89)

*Note:* Values are presented as mean ± standard deviation and median (minimum–maximum). Lambda_z: terminal phase rate constant; *T*½: half‐life; CMax: maximum plasma concentration; C0: plasma concentration at time zero; TMax: time to reach maximum plasma concentration; *AUC*_0→*t*_: area under the curve from time zero to the last measurable concentration; *AUC*_0→∞_: area under the curve from time zero to infinity; *AUC*_0→*t*_/*AUC*_0→∞_: ratio of *AUC*_0→*t*_ to *AUC*_0→∞_; *MRT*_0→∞_: mean residence time from time zero to infinity; Vd: apparent volume of distribution; Cl: total body clearance.

* Significant difference.

Table 2 presents the results for the metabolite AA. Statistically significant differences between G1 and G2 were observed for *AUC*_0→*t*_, *AUC*_0→∞_, *MRT*_0→∞_ and Cl.

**TABLE 2:** Pharmacokinetic parameters of 4‐aminoantipyrine (AA) following intravenous administration of 25 mg kg^−1^ dipyrone (G1) and 25 mg kg^−1^ dipyrone combined with 2 mg kg^−1^ tramadol (G2) in dogs (*n* = 9).

AA		G1 Dipyrone 25 mg kg^−1^		G2 Dipyrone 25 mg kg^−1^ + Tramadol 2 mg kg^−1^	
Parameters	Unit	Mean ± SD	Median (min–max)	Mean ± SD	Median (min–max)
Lambda_z (*λz*)	1/h	0.11 ± 0.03	0.11 (0.08–0.15)	0.11 ± 0.03	0.10 (0.07–0.17)
*T*½	h	6.43 ± 1.63	6.23 (4.37–8.25)	6.82 ± 1.71	6.81 (4.04–9.02)
Cmax	μg/mL	2.75 ± 1.52	2.56 (1.10–5.50)	3.48 ± 0.65	3.64 (2.46–4.41)
C0	μg/mL	—			
Tmax	h	6.44 ± 2.40	6.00 (4.00–10)	6.61 ± 3.41	6.00 (1.5–12)
*AUC*_0→*t*_	μg/mL h	42.52 ± 20.61*	41.92 (12.77–79.02)	67.71 ± 20.63*	61.30 (44.03–109.36)
*AUC*_0→∞_	μg/mL h	43.23 ± 20.56*	42.66 (13.37–79.65)	69.01 ± 21.51*	62.20 (45.40–113.17)
*AUC*_0→*t*_/_0→∞_		0.98 ± 0.02	0.98 (0.95–0.99)	0.98 ± 0.01	0.98 (0.96–0.99)
*MRT*_0→∞_	h	11.79 ± 2.87*	13.15 (6.90–14.83)	14.50 ± 2.20*	15.13 (10.78–18.02)
Vd	(L)	460.85 ± 272.43	335.07 (187.91–843.82)	255.72 ± 105.89	221.65 (166.70–477.85)
Cl	(L/h)	50.67 ± 32.72*	39.07 (20.92–124.60)	26.07 ± 7.20*	26.79 (14.72–36.71)

*Note:* Values are presented as mean ± standard deviation and median (minimum–maximum). Lambda_z: terminal phase rate constant; *T*½: half‐life; CMax: maximum plasma concentration; C0: plasma concentration at time zero; TMax: time to reach maximum plasma concentration; *AUC*_0→*t*_: area under the curve from time zero to the last measurable concentration; *AUC*_0→∞_: area under the curve from time zero to infinity; *AUC*_0→*t*_/*AUC*_0→∞_: ratio of *AUC*_0→*t*_ to *AUC*_0→∞_; *MRT*_0→∞_: mean residence time from time zero to infinity; Vd: apparent volume of distribution; Cl: total body clearance.

* Significant difference.

The pharmacokinetic parameters of AA that showed statistical differences were evaluated for linear correlation. A Pearson linear correlation was observed between AUC and Cl, showing a strong negative correlation (*R* ^2^ = −0.81; *p* < 0.001; Cl = −7.7 × 10^−10^ × *AUC*_0–*t*_ + 2.93 × 10^−4^).

A violin plot (Figure 5) was generated to represent the AA metabolite variables that exhibited statistically significant differences.

Table 3 presents the descriptive pharmacokinetic parameters of tramadol and a comparison with selected studies reported in the literature.

**TABLE 3:** Pharmacokinetic parameters of tramadol following intravenous administration of 2 mg kg^−1^ tramadol combined with 25 mg kg^−1^ dipyrone (*n* = 9) in dogs.

G2 Tramadol		Dipyrone 25 mg kg^−1^ + Tramadol 2 mg kg^−1^		Kukanich and Papich (2004); 4.4 mg kg^−1^	McMillan et al. (2008) 2 mg kg^−1^	Vettorato et al. (2010) 2 mg kg^−1^	Giorgi et al. (2010) 4 mg kg^−1^
Parameters	Unit	Mean ± SD	Median (min–max)	Mean ± SD	Mean ± SD	Mean ± SD	Mean ± SD
Lambda_z (*λz*)	1/h	0.11 ± 0.08	0.09 (0.02–0.28)	0.89 ± 0.13	—	—	—
*T*½	H	10.30 ± 8.30	8.05 (2.42–31.13)	0.80 ± 0.12	1.04 ± 0.42	2.24 ± 0.87	0.41 ± 0.39
Cmax	μg/mL	1.11 ± 0.42	1.07 (0.57–1.97)	—	—	—	—
C0	μg/mL	0.32 ± 0.69	0.010 (0.004–2.00)	0.77 ± 0.18	—	5.48 ± 5.92	—
Tmax	H	—	—	—	—	—	—
*AUC*_0→*t*_	μg/mL*h	2.17 ± 0.96	1.90 (1.11–3.92)	—	0.82 ± 0.29	—	—
*AUC*_0→∞_	μg/mL*h	2.25 ± 0.95	2.00 (1.14–3.96)	0.13 ± 0.02	—	3.56 ± 0.67	2.16 ± 0.79
*AUC*_0→*t*/0→∞_		0.96 ± 0.04	0.98 (0.84–0.99)	—	—	—	—
*MRT*_0→∞_	H	4.97 ± 3.16	3.98 (2.65–13.53)	0.93 ± 0.12	—	2.63 ± 1.13	1.13 ± 1.13
Vd	(L)	1027.89 ± 810.67	977.16 (217.08–2982.10)	0.3 ± 0.04	0.05 ± 0.01	9.56 ± 5.17	0.11 ± 0.03
Cl	(L/h)	69.89 ± 29.74	66.40 (33.63–116.46)	49.17 ± 7.37	40.61 ± 14.03	26.23 ± 18.60	13.85 ± 6.90

*Note:* Values are presented as mean ± standard deviation and median (minimum–maximum). Comparison with previously published studies (Kukanich and Papich 2004; McMillan et al. 2008; Vettorato et al. 2010; Giorgi et al. 2010) (Dose‐dependent values were adjusted to 2 mg kg^−1^ for comparison). Lambda_z: terminal phase rate constant; *T*½: half‐life; CMax: maximum plasma concentration; C0: plasma concentration at time zero; TMax: time to reach maximum plasma concentration; *AUC*_0→*t*_: area under the curve from time zero to the last measurable concentration; *AUC*_0→∞_: area under the curve from time zero to infinity; *AUC*_0→*t*_/*AUC*_0→∞_: ratio of *AUC*_0→*t*_ to *AUC*_0→∞_; *MRT*_0→∞_: mean residence time from time zero to infinity; Vd: apparent volume of distribution; Cl: total body clearance.

Table 4 presents the descriptive pharmacokinetic parameters of the M1 metabolite and a comparison with selected studies reported in the literature.

**TABLE 4:** Pharmacokinetic parameters of O‐desmethyltramadol (M1) following intravenous administration of 2 mg kg^−1^ tramadol combined with 25 mg kg^−1^ dipyrone (*n* = 9) in dogs.

Parameters	Unit	M1		Vettorato et al. (2010) 2 mg kg^−1^	Giorgi et al. (2010) 4 mg kg^−1^
		Mean ± SD	Median (min–max)	Mean ± SD	Mean ± SD
Lambda_z (*λz*)	1/h	0.09 ± 0.05	0.06 (0.02–0.15)	3.59 ± 0.88	0.52
*T*½	h	11.55 ± 8.07	11.01 (4.52–28.66)		1.34
Cmax	μg/mL	0.012 ± 0.006	0.012 (0.003–0.02)	0.35 ± 0.17	0.011 ± 0.002
C0	μg/mL	—			
Tmax	h	0.90 ± 0.56	0.75 (0.25–1.5)	0.29 ± 0.16	1.70 ± 1.34
*AUC*_0→*t*_	μg/mL*h	0.06 ± 0.03	0.05 (0.03–0.14)		
*AUC*_0→∞_	μg/mL*h	0.06 ± 0.03	0.05 (0.03–0.16)	1.24 ± 0.16	0.013
*AUC*_0→*t*_/_0→∞_		0.92 ± 0.08	0.94 (0.77–0.99)		
*MRT*_0→∞_	h	11.46 ± 5.59	9.80 (4.48–21.84)	5.42 ± 2.54	2.18
Vd	(L)	35147.95 ± 20156.35	27287.94 (16152.93–67157.48)	—	—
Cl	(L/h)	2411.58 ± 909.93	2551.15 (825.46–3762.00)	—	—

*Note:* Values are presented as mean ± standard deviation and median (minimum–maximum). Comparison with previously published studies (Vettorato et al. 2010; Giorgi et al. 2010). (Dose‐dependent values were adjusted to 2 mg kg^−1^ for comparison). Lambda_z: terminal phase rate constant; *T*½: half‐life; CMax: maximum plasma concentration; C0: plasma concentration at time zero; TMax: time to reach maximum plasma concentration; *AUC*_0→*t*_: area under the curve from time zero to the last measurable concentration; *AUC*_0→∞_: area under the curve from time zero to infinity; *AUC*_0→*t*_/*AUC*_0→∞_: ratio of *AUC*_0→*t*_ to *AUC*_0→∞_; *MRT*_0→∞_: mean residence time from time zero to infinity; Vd: apparent volume of distribution; Cl: total body clearance.

Table 5 presents the descriptive pharmacokinetic parameters of the M2 and M5 metabolites and a comparison with selected studies reported in the literature.

**TABLE 5:** Pharmacokinetic parameters of N‐desmethyltramadol (M2) and N,O‐desmethyltramadol (M5) following intravenous administration of 2 mg kg^−1^ tramadol combined with 25 mg kg^−1^ dipyrone (*n* = 9) in dogs.

G2	Dipyrone 25 mg kg^−1^ + Tramadol 2 mg kg^−1^	M2		Giorgi et al. (2010) 4 mg kg^−1^	M5		Giorgi et al. (2010) 4 mg kg^−1^
Parameters	Unit	Mean ± SD	Median (min–max)	Mean ± SD	Mean ± SD	Median (min–max)	Mean ± SD
Lambda_z (*λz*)	1/h	0.12 ± 0.11	0.07 (0.04–0.38)	0.27 ± 0.07	0.14 ± 0.07	0.12 (0.08–0.28)	0.26 ± 0.07
*T*½	h	8.78 ± 4.83	9.46 (1.80–15.83)	2.63 ± 0.55	5.56 ± 1.89	5.95 (2.41–7.79)	2.79 ± 0.70
Cmax	μg/mL	0.006 ± 0.003	0.006 (0.002–0,01)	0.17 ± 0.05	0.001 ± 0.001	0.001 (0.0006–0.007)	0.19 ± 0.02
C0	μg/mL	—			—		
Tmax	h	1.40 ± 0.58	1.50 (0.25–1.5)	0.0004 ± 0.0002	1.33 ± 0.43	1.50 (0.50–2.00)	0.0004 ± 0.0003
*AUC*_0→*t*_	μg/mL*h	0.03 ± 0.01	0.03 (0.01–0.05)	—	0.006 ± 0.003	0.004 (0.003–0.01)	
*AUC*_0→∞_	μg/mL*h	0.03 ± 0.01	0.03 (0.01–0.05)	0.59 ± 0.21	0.006 ± 0.004	0.004 (0.003–0.014)	0.78 ± 0.15
*AUC*_0→*t*_/_0→∞_		0.98 ± 0.01	0.99 (0.96–0,99)		0.96 ± 0.02	0.96 (0.93–0.99)	
*MRT*_0→∞_	h	5.75 ± 1.55	5.83 (3.12–8.88)	3.83 ± 0.76	5.36 ± 1.54	6.09 (2.84–7.35)	4.32 ± 0.72
Vd	(L)	52058.27 ± 30217.92	39051.15 (22223.26–98895.48)	—	209180.65 ± 102742.84	184715.36 (48255.32–359925.50)	—
Cl	(L/h)	10154.60 ± 9452.40	4655.25 (2428.75–49923.63)	—	25500.51 ± 11313.00	27046.58 (9120.14–43542.08)	—

*Note:* Values are presented as mean ± standard deviation and median (minimum–maximum) (Giorgi et al. 2010). Lambda_z: terminal phase rate constant; *T*½: half‐life; CMax: maximum plasma concentration; C0: plasma concentration at time zero; TMax: time to reach maximum plasma concentration; *AUC*_0→*t*_: area under the curve from time zero to the last measurable concentration; *AUC*_0→∞_: area under the curve from time zero to infinity; *AUC*_0→*t*_/*AUC*_0→∞_: ratio of *AUC*_0→*t*_ to *AUC*_0→∞_; *MRT*_0→∞_: mean residence time from time zero to infinity; Vd: apparent volume of distribution; Cl: total body clearance.

Regarding behavioral and adverse effects, in G1, three animals exhibited tachypnea, while in G2, three animals showed nausea and one exhibited hypersalivation, occurring shortly after the start of drug administration, with a mean duration of 15 min in both groups.

## 4 Discussion

To our knowledge, this is the first study to characterize the pharmacokinetic profile of dipyrone administered in combination with tramadol in dogs following intravenous administration. This drug combination is widely used in clinical practice in Brazil as part of multimodal analgesic protocols for dogs, as reported in national surveys and clinical studies (Lorena et al. 2014; Flôr et al. 2013; Teixeira et al. 2013). It was possible to quantify the metabolites of both compounds.

Differences between the present findings and previously published studies may be explained by variations in species, study design, dosing regimen, and analytical methodology. In rats, the dipyrone–tramadol combination has primarily been evaluated under experimental pain or inflammatory models and/or repeated dosing conditions, often including pharmacodynamic endpoints, and the magnitude of pharmacokinetic changes has been reported to differ between single and multiple administrations (Lopez‐Muñoz et al. 2013; Moreno‐Rocha et al. 2016). In contrast, the present study employed a two‐period crossover design with a 15‐day washout and assessed a single intravenous administration in healthy dogs, focusing exclusively on pharmacokinetic outcomes. In addition, interspecies metabolic variability is well recognized and may contribute to discrepant results, as suggested by studies using comparable doses and analytical methodologies in other species, such as donkeys (Araújo‐Silva et al. 2024). Furthermore, tramadol pharmacokinetics in dogs following intravenous administration have shown substantial interstudy variability, particularly regarding metabolite formation and systemic exposure (Kukanich and Papich 2004; McMillan et al. 2008; Giorgi et al. 2010). Therefore, cross‐study comparisons should be interpreted cautiously, taking into account these methodological and biological differences.

Regarding the available pharmacokinetic literature on the dipyrone‐tramadol co‐administration, studies have been conducted only in rats and donkeys. Lopez‐Muñoz et al. (2013) investigated different combinations of dipyrone and tramadol doses (subcutaneously) in rats with arthritis, using single administrations and evaluating antinociceptive effects and dose–response curves. Results showed that the combination produced additive or potentiating effects, and the authors suggested a possible pharmacokinetic interaction between the drugs, although the nature of this interaction was not detailed. They proposed that the drugs could compete for the same cytochrome P450 enzymes (glucuronidation pathway), leading to alterations in metabolite concentrations and pharmacological effects, suggesting further studies.

In this context, Moreno‐Rocha et al. (2016) used one of the previously tested dose combinations to analyze the pharmacokinetics and pharmacodynamics of dipyrone alone and in combination with tramadol in rats with induced arthritis. The authors found no significant differences in pharmacokinetic parameters for MAA or AA following a single administration between treatments, indicating that tramadol did not alter the pharmacokinetics of dipyrone metabolites when co‐administered in a single dose. Significant differences were observed only after multiple daily doses (4 consecutive days), suggesting the need for additional studies with multiple‐dose regimens in other species.

Although Moreno‐Rocha et al. (2016) reported no significant pharmacokinetic differences after a single administration, a trend toward lower MAA Cmax was observed when dipyrone was combined with tramadol. In the present study, this reduction was significant, with Cmax and C0 markedly lower in the combination group. These findings indicate a pharmacokinetic interaction occurring at an early stage of drug disposition.

Considering the intravenous route and the rapid conversion of dipyrone to MAA, the reduction in peak concentrations is more consistent with alterations in the initial appearance of MAA in the systemic circulation rather than changes in overall exposure, as AUC remained unchanged. This profile suggests a redistribution of exposure over time, which may contribute to a lower likelihood of acute concentration‐dependent adverse effects without compromising sustained MAA plasma levels.

Araújo‐Silva et al. (2024) conducted a pharmacokinetic study of dipyrone alone and in combination with tramadol (intravenous) in donkeys using similar doses and the same analytical methodology and instrumentation as the present study. For MAA, results indicated a (non‐significant) increase in *T*½ when the drugs were combined. In the present study, although the increase was not significant, *T*½ also increased with the combination. It is important to note that this study continues from a previous publication (Mouta et al. 2025), which identified differences between ultrarapid metabolizers and normal/rapid metabolizers. The variation in dipyrone metabolism between rapid and slow metabolizers influenced tramadol metabolism, leading to the observed variations in T ½, Cmáx, AUC and Cl. The mean values in the present study were described based on all animals, without distinguishing metabolizer groups.

Regarding MAA, a slight reduction in AUC was observed in G2, without significant differences, suggesting that the combination did not alter the systemic exposure to dipyrone. The minimum plasma concentrations of MAA required to inhibit 50% of cyclooxygenase activity (IC50—COX‐1 and COX‐2) have been reported in humans, with values of 0.553 μg/mL for IC50‐COX1 and 0.926 μg/mL for IC50‐COX2 (Hinz et al. 2007; Knights et al. 2010). In G1, three animals maintained plasma concentrations above these values for 48 h, whereas in G2, six animals exceeded these thresholds. These results indicate that dipyrone alone or in combination with tramadol achieved the minimum inhibitory concentrations of COX‐1 and COX‐2 for MAA.

Regarding the AA metabolite, *AUC*_0→*t*_ and *AUC*_0→∞_ increased significantly in G2, indicating increased systemic exposure to AA. A significant reduction in Cl was also observed, suggesting decreased elimination. The observed reduction in AA clearance may reflect an interaction at the metabolic level, potentially involving shared CYP‐mediated pathways. Nevertheless, mechanistic studies evaluating enzymatic activity would be necessary to confirm this hypothesis. This pharmacokinetic interaction should be evaluated in future studies using multiple‐dose regimens, as it may be relevant during prolonged treatment and could differ between single‐ and multiple‐dose administrations (Moreno‐Rocha et al. 2016). *MRT*_0→∞_ of AA also increased significantly in G2, suggesting that the metabolite remained longer in circulation due to the reduced Cl.

In the previously mentioned rat study, no animals exhibited adverse effects resulting from the drug combination. In donkeys, adverse effects such as restlessness and ataxia were observed in two animals (Araújo‐Silva et al. 2024), and in the present study, some animals showed nausea and hypersalivation with the drug combination. These signs have been previously reported in other studies using tramadol in different species, including donkeys (Mouta et al. 2021) and horses (Knych et al. 2013).

Although a group receiving tramadol alone was not included, the pharmacokinetic profile of this agent and its main metabolites when combined with dipyrone was described in this study and compared with previous reports (Tables 3, 4, 5). According to Domínguez‐Oliva et al. (2021), dogs produce higher concentrations of M2 than M1. However, in the present study, M1 concentrations were higher than those of M2 and M5, which differs from previously reported intravenous tramadol studies in dogs (Giorgi et al. 2010). This difference may reflect potential competition promoted by the combination, specifically along the N‐demethylation pathway, resulting in reduced formation of M2 and M5. MAA is converted to AA via CYP3A4 and CYP2B6, which may reduce M2 formation and favor O‐demethylation, thereby also limiting M5 production. These findings are supported by the lower observed M5 concentrations. Dogs produce M2 primarily through CYP2B11 (Perez‐Jimenez et al. 2016), which may be homologous to the CYP enzymes involved in dipyrone metabolism.

Indirect quantification of metabolites using LC‐MS/MS in the absence of pure standards is feasible but requires methodological caution and validation (Ghafari and Sleno 2024). The use of calibration curves from structurally related compounds can provide conceptual support, although analytical responses may vary among metabolites; therefore, results should be interpreted as approximate estimates (Kruve 2020). In the specific case of tramadol, although there is no direct evidence for using the M1 calibration curve to quantify M2 and M5, the literature suggests that this approach is viable if the method is validated, including assessment of accuracy and precision. Thus, the use of the M1 calibration curve as a substitute for M2 and M5 should be accompanied by validation experiments to confirm applicability and minimize errors arising from differences in ionization responses and matrix effects. Regarding tramadol, compared to previous studies, reductions in C0 and increases in MRT and *T*½ were observed. These findings may indicate a distributive interaction. The reduction in C0 suggests that the combined drug was distributed more rapidly into tissues, lowering initial plasma concentration. Increases in MRT and *T*½ may indicate that the drug redistributed from tissues back into circulation slowly. A metabolic interaction at the binding site may also have occurred, with the increase suggesting that dipyrone inhibited tramadol metabolism, prolonging its presence in the organism.

For M1, an increase in *T*½ and a reduction in AUC were observed when co‐administered with dipyrone. This suggests that combining dipyrone with tramadol may not be advantageous if the goal is to increase plasma concentrations of M1. Notably, tramadol concentrations increased substantially. Future studies evaluating tramadol's analgesic profile in dogs are necessary to determine whether analgesia derives primarily from M1 (the active metabolite), as in other species, or from the parent drug.

The minimum effective concentration (MEC) for analgesia of tramadol and M1 has not yet been described in dogs; however, in humans, MEC of tramadol was found to range from 298 (127–469) ng/mL (Lehmann et al. 1990) to 590 (180–1000) ng/mL (Grond et al. 1999), and MEC of M1 was found to range from 39.6 (10.1–69.1) ng/mL (Lehmann et al. 1990) to 84 (50–134) ng/mL (Grond et al. 1999). If these concentrations apply to dogs, in the present study, the Cmáx for tramadol was substantially higher than the MEC established for humans, suggesting that therapeutic levels were achieved. However, the Cmáx for M1 was remaining below the human therapeutic range. This indicates that the analgesic effect in these animals may be primarily driven by the parent drug rather than the M1 metabolite, or that the species‐specific MEC for M1 differs from that of humans. Of course, species‐specific analgesiometric testing is ultimately needed in order to assess tramadol and M1 efficacy in dogs.

Regarding the clinical efficacy of tramadol, current evidence and international guidelines support its role within multimodal analgesic strategies, despite its recognized pharmacokinetic variability in the canine species. The 2022 AAHA Pain Management Guidelines categorize tramadol as an adjunctive therapy for acute pain, emphasizing that it should be part of a tiered, multimodal approach rather than a standalone treatment (Gruen et al. 2022). Similarly, the 2022 WSAVA guidelines include tramadol in their analgesic protocols, particularly in scenarios where access to full μ‐opioid agonists is restricted, reinforcing its importance in global veterinary practice (Monteiro et al. 2022). Furthermore, a systematic review and meta‐analysis by Donati et al. (2021) demonstrated that tramadol administration likely reduces the need for rescue analgesia compared to placebo in postoperative settings. While the metabolic profile described in the present study reflects the known individual variation in dogs, these international references justify the clinical use of tramadol as a viable component for managing acute pain in Brazil.

The doses used in the present study were based on clinical usage and previous studies. The same doses were applied by Flôr et al. (2013), who evaluated dipyrone/tramadol, tramadol alone, and dipyrone/carprofen combinations for chronic oncologic pain control over 14 days using pain scales and quality‐of‐life assessments. They reported that the dipyrone/tramadol combination was effective and safe, and that adding an anti‐inflammatory agent did not confer additional benefit in most animals.

Teixeira et al. (2013) evaluated a combination of tramadol (3 mg kg^−1^) and dipyrone (30 mg kg^−1^) intravenously. Analgesic efficacy was assessed in the immediate postoperative period in bitches undergoing unilateral mastectomy with or without ovariohysterectomy. The combination provided effective analgesia for up to 24 h, with pain scores comparable to groups treated with tramadol alone or tramadol combined with meloxicam. In the tramadol/dipyrone combination, pain scores remained above baseline for up to 8 h, and the number of animals requiring rescue analgesia was slightly lower than in the tramadol‐alone group, although no significant differences were observed. These clinical studies support the rationale for selecting these agents in the present research.

The limitations of this study include the absence of a tramadol‐only control group for direct pharmacokinetic comparison and the indirect quantification of the M2 and M5 metabolites, which was required due to the unavailability of analytical reference standards. In addition, the study was conducted exclusively in clinically healthy dogs, which may limit the extrapolation of the results to clinical populations with altered physiological or pathological conditions. Therefore, further studies are warranted to characterize the pharmacokinetic behavior of tramadol and its metabolites under different clinical scenarios, including multiple‐dose regimens, while interpretations related to analgesic efficacy should remain outside the scope of the present pharmacokinetic investigation.

## 5 Conclusion

A significant pharmacokinetic interaction exists between dipyrone and tramadol, reflected by reduced elimination and increased AA AUC. These findings suggest that the combination may alter drug exposure, potentially influencing the duration and intensity of analgesia. However, as the most severe adverse effects of dipyrone are primarily attributed to MAA rather than AA, the direct clinical implications for safety should be interpreted with caution. Additionally, inhibition of tramadol conversion to M1 was observed.

## Author Contributions

**Andressa Nunes Mouta:** data acquisition, preparation of the original work. **Kathryn Nóbrega Arcoverde**, **Robson dos Anjos Honorato**, **Naftáli Silva Fernandes**, **Yanna Deysi Bandeira Passos**, **Caio Vinicius Almeida de Oliveira:** data acquisition. **Gabriel Araújo‐Silva**, **Valéria Veras de Paula:** developed the applied methodology, performed the statistical analyses, supervised the work and reviewed the final manuscript.

## Funding

The present work was conducted with financial support from CNPq, the National Council for Scientific and Technological Development, Brazil, within the scope of the Universal Program (403419/2023‐9).

## Conflicts of Interest

The authors declare no conflicts of interest.

## Data Availability

The data are not publicly available due to ethical and institutional restrictions but are available from the corresponding author upon reasonable request.
